# Lack of impact of the El Hierro (Canary Islands) submarine volcanic eruption on the local phytoplankton community

**DOI:** 10.1038/s41598-018-22967-6

**Published:** 2018-03-16

**Authors:** M. Gómez-Letona, J. Arístegui, A. G. Ramos, M. F. Montero, J. Coca

**Affiliations:** 1 Instituto de Oceanografía y Cambio Global, IOCAG, Universidad de Las Palmas de Gran Canaria, ULPGC, Las Palmas de Gran Canaria, Las Palmas, Spain; 2 División de Robótica y Oceanografía Computacional, IUSIANI, Universidad de Las Palmas de Gran Canaria, ULPGC, Las Palmas de Gran Canaria, Las Palmas, Spain

## Abstract

The eruption of a submarine volcano south of El Hierro Island (Canary Islands) in October 2011 led to major physical and chemical changes in the local environment. Large amounts of nutrients were found at specific depths in the water column above the volcano associated with suboxic layers resulting from the oxidation of reduced chemical species expelled during the eruptive phase. It has been suggested that the fertilization with these compounds enabled the rapid restoration of the ecosystem in the marine reserve south of the island once the volcanic activity ceased, although no biological evidence for this has been provided yet. To test the biological fertilization hypothesis on the pelagic ecosystem, we studied the evolution and variability in chlorophyll *a*, from *in situ* and remote sensing data, combined with information on phytoplankton and bacterial community structure during and after the eruptive episode. Remote sensing and *in situ* data revealed that no phytoplankton bloom took place neither during nor after the eruptive episode. We hypothesize that the fertilization by the volcano did not have an effect in the phytoplankton community due to the strong dilution of macro- and micronutrients caused by the efficient renewal of ambient waters in the zone.

## Introduction

**Figure 1:**
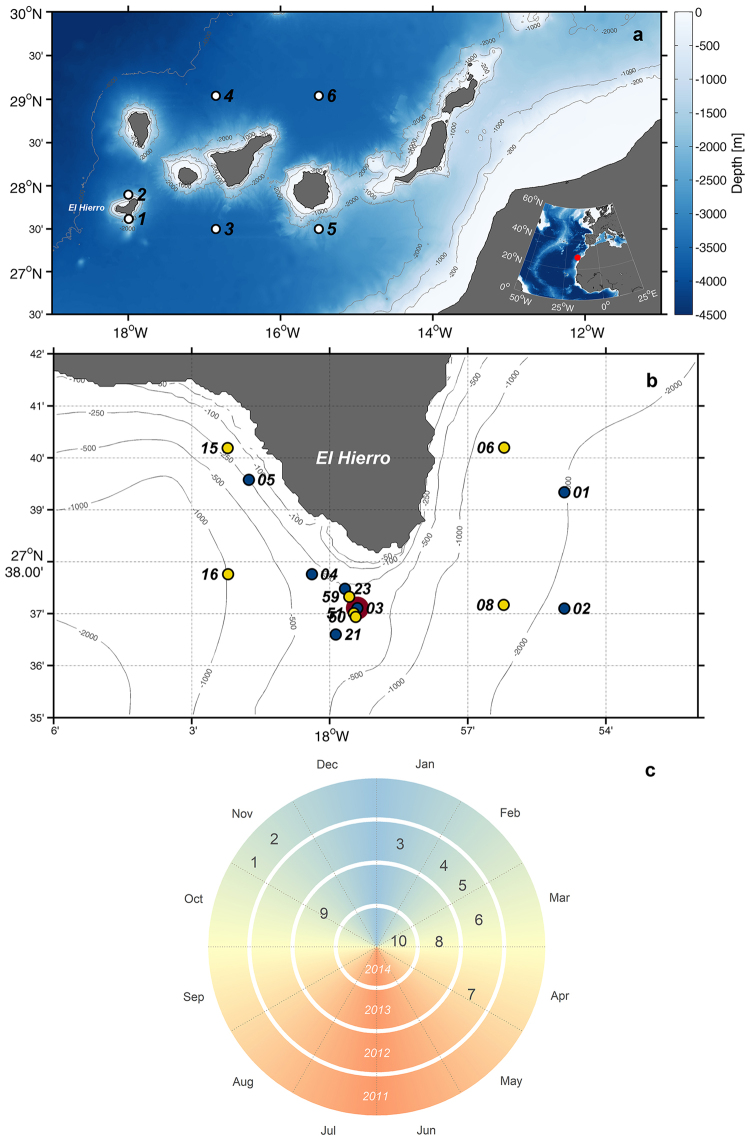
(**a**) Location of El Hierro within the Canary Islands archipelago. 1–6 are the points where remote sensing Chl-a was analysed. (**b**) Hydrographic stations along the period of study. Dark blue, cruises 1‒5; yellow, cruises 8‒10; large, burgundy circle indicates the location of the volcano. Stations from the two separate set of cruises are paired as follows: 01 & 06; 02 & 08; 03 & 51; 04 & 16; 05 & 15; 21 & 50; 23 & 59. (**c**) Temporal distribution of cruises. Maps were generated using Matlab 7.14 R2012a (https://www.mathworks.com/products/matlab).

After more than 11,000 earthquakes and anomalous gas emissions, a submarine eruption took place at the leeward side of El Hierro Island (Canary Islands, Fig. 1a), near a marine reserve, on October 10, 2011. At this time, the eruption site was located 5 km off the coast at a depth of 900 m, but migrated northwards during the first three days, ceasing its advance 1.8 km off the coast^1^. On late October a first bathymetric survey showed a 100 m-high volcanic cone at 350 m depth. A subsequent survey performed on early December reflected that the original edifice had evolved into one formed by three similarly sized cones with their summits at 180–160 m depth^2^. The eruptive phase peaked during late October and early November, diminishing in intensity the weeks after, and ceasing completely by late February, 2012^1,3^ ‒when the main cone was situated at 88 m depth‒ giving place to a degasification stage that remains active^4^.

During the five month period (October, 2011 – February, 2012) in which the eruptive phase took place, abundant materials were expelled to the water column, greatly altering its physical and chemical properties^3–5^. These variations were not constant throughout the eruptive phase and depended on the spatial proximity to the emission zone, both horizontally and vertically. An increase in temperature and a decrease in salinity were accompanied by a discoloration of seawater associated with the emission of high-temperature hydrothermal fluids, magmatic gases and volcanic particles that reached the ocean surface. The bubbling and degassing related to the eruption caused the input of CO_2_, greatly augmenting its partial pressure (pCO_2_) and the total inorganic carbon (C_T_) concentration, and markedly decreasing the alkalinity (A_T_) and pH levels^3,5,6^. Emissions of Fe(II) and reduced species of sulphur provoked a fall in the redox potential, even reaching negative values at certain depths. The oxidation of those species resulted in very low concentrations of dissolved O_2_, eventually reaching anoxic conditions in subsurface waters, with major implications for the biota.

Acoustic surveys confirmed the absence of fish schools in the area affected by the volcano, while many dead individuals were observed in the surface, due to the anoxic or suboxic conditions of the subsurface waters^3^. It was observed^7^ that the mesopelagic zooplankton and fish Migrant Scattering Layer (MSL) was weaker and the upper limit of the Diel Vertical Migration (DVM) was shallower below the oxygen minimum zone, both anomalies being associated with an increase in surface turbidity and a dramatic decrease in dissolved O_2_. On the contrary, high nucleic acid (HNA)-content bacteria (the most active populations) experienced an increase in their abundance linked to the environmental changes generated by the eruption. These alterations were however only temporal, and the typical conditions for the microbial communities were restored by January–February, 2012^8^.

Elevated concentrations of inorganic macro-nutrients (nitrate, NO_3_^−^ + NO_2_^−^; phosphate, PO_4_^3−^; and silicate, Si(OH)_4_) and iron -Fe (II)- were reported^5^ at station 3 (Fig. 1b) above the volcano, during the eruptive phase (November 2011). Maximum values were registered at about 75–100 m depth, in the upper thermocline, associated with the suboxic layer^3^, during November, 2011, with Fe(II), nitrate, phosphate and silicate concentrations reaching, respectively, 50, 8, 0.5 and 23 μmol·kg^−1^. After the eruptive phase, nutrient concentrations at this station followed a more coherent pattern with the annual cycle reported for the Canary Islands waters^9^, with nitrate concentrations ~1 μmol·kg^−1^ in the surface waters due to winter mixing (February 2012)^3^. In spite of the very localized increases of nutrients associated with oxygen minimum layers and the lack of data as to their extension, it was suggested^5^ that the input of nutrients to the euphotic zone by the volcanic activity would act as a biological fertilizer, providing the ingredients for the fast recovery of the ecosystem south of the island, once the eruption ceased.

In order to test the hypothesis of nutrient fertilization on primary producers –that would lead to a fast ecosystem restoration after the volcanic eruption–, we studied the distribution of the phytoplankton community at stations affected and non-affected by the volcanic emissions by looking at the chlorophyll concentration and the phytoplankton community structure, along 10 cruises, spanning both the eruptive and post-eruptive phases, from October 2011 to March 2014 (Fig. 1c; Supplementary Table S1). Furthermore, *in situ* measurements have been complemented with remote sensing chlorophyll estimates to place in context the seasonal evolution of our measurements at a regional and inter-annual scale.

## Results

### *In situ* Chlorohyll-a (Chl-a)

**Figure 2:**
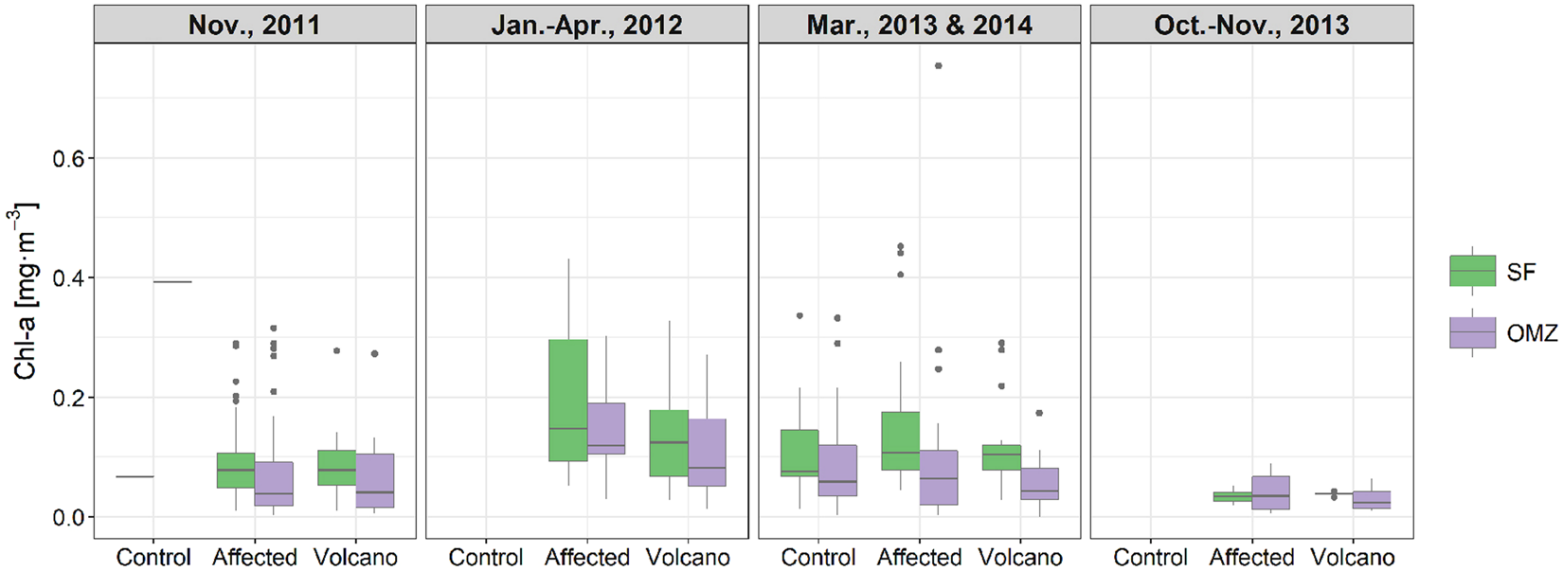
*In situ* Chl-a concentrations (mg·m^−3^). Periods: Nov., 2011 (cruises 1 and 2), Jan.–Apr., 2012 (cruises 3‒7), Mar., 2013 and 2014 (cruises 8 and 10), Oct.–Nov., 2013 (cruise 9). Locations: control (control stations), volcano (stations near the volcano) and affected (any other station affected by the eruption). Depths: SF (subsurface waters, 0–70 m) and OMZ (oxygen minimum zone waters, 70–200 m). In the boxes, the lower and upper hinges correspond to the 25th and 75th percentiles. The upper (lower) whisker extends from the hinge to the largest (smallest) value no further than 1.5 * IQR from the hinge, where IQR is the inter-quartile range, i.e., the distance between the 25th and 75th percentiles. Data beyond the end of the whiskers are plotted individually.

Overall, average annual concentrations of Chl-a (Fig. 2a) estimated from discrete seawater samples were low, usually ranging between 0.05–0.3 mg·m^−3^, rarely exceeding these values. There was little variability between average values in the upper 70 m (SF) and the oxygen minimum zone waters (70–200 m; OMZ), probably because the deep chlorophyll maximum (DCM) was usually located below the 70 m depth boundary. There were no statistical differences between stations affected or not by the volcano emissions (control/affected/volcano; Fig. 1b; Supplementary Table S1), both at the SF and OMZ, during a same sampling period (Fig. 2a). Cruises 1–2 (Nov., 2011) and 9 (Oct.-Nov., 2013) showed, however, lower concentrations, compared with 3–7 (Jan.–Apr., 2012), and 8 and 10 (Mar., 2013 and 2014). In fact, differences were significant (p ≤ 0.01) in SF between the autumn and winter/spring periods, whereas the concentrations did not change significantly in OMZ waters.

**Figure 3:**
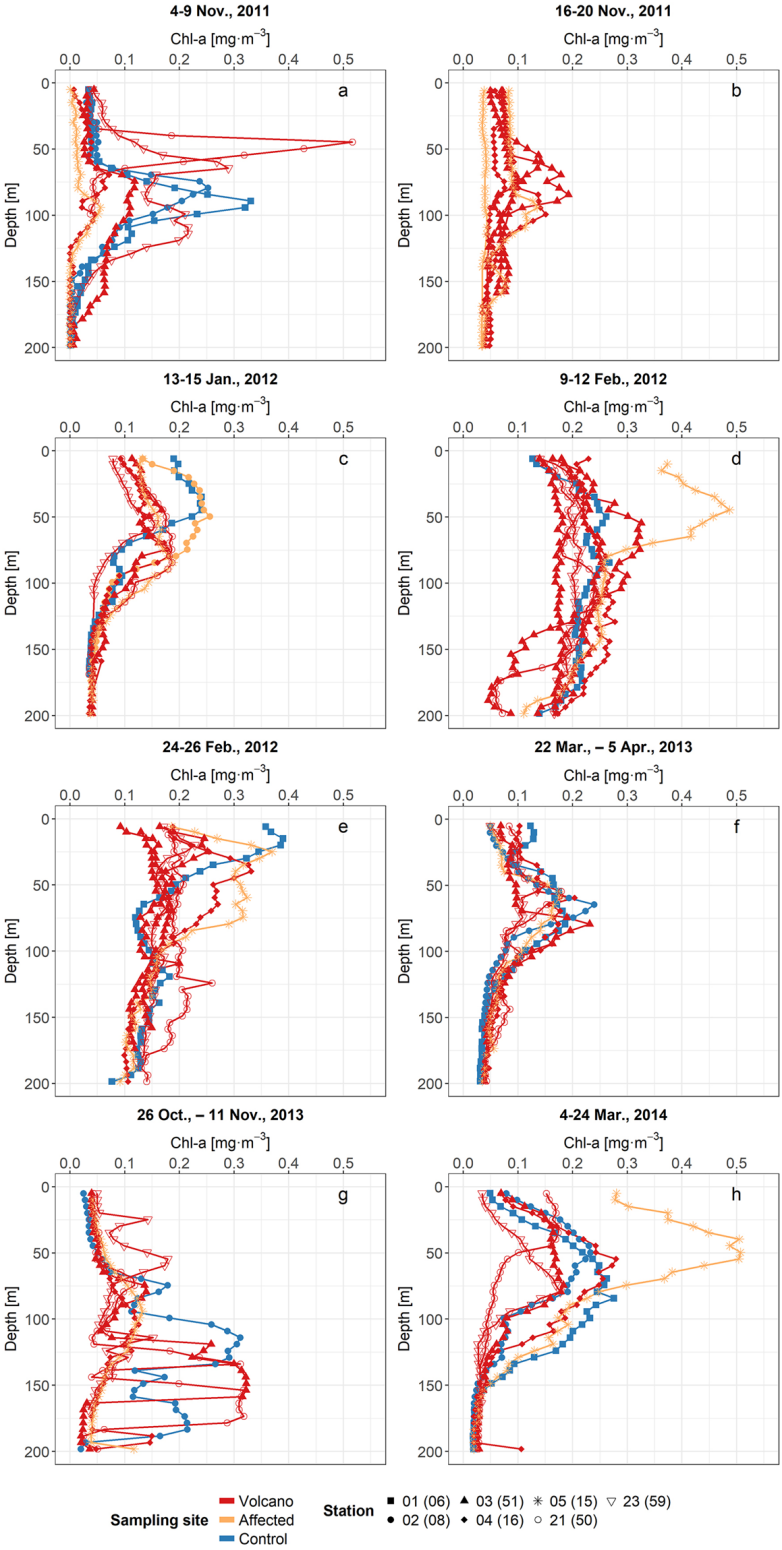
Chl-a profiles derived from CTD-fluorometer. Cruises 1(a)–5(e) and 8(f)–10(h). For cruises 8‒10 station correspondence with cruises 1‒5 is indicated in brackets (see Fig. 1b).

Profiles of Chl-a derived from the CTD-fluorometer (Fig. 3) showed temporal and vertical variability between the various cruises. As observed with *in situ* discrete measurements, average Chl-a in profiles was higher in the water column during the late-winter/spring period (cruises 4, 5 and 10). However, there were also differences between stations during a same cruise. For instance, station 5(15), which was located over the narrow island shelf, exhibited contrasting high Chl-a during cruises 4, 5 and 10. Narrow subsurface and deep peaks of fluorescence Chl-a were apparent at stations around the volcano, during 4‒9 Nov., 2011; however, these anomalously high peaks were not due to actual Chl-a but to coloured materials expelled during the eruption, which interfered with fluorescence measurements. *In situ* Chl-a derived from extracted pigments at the same depths confirmed that actual concentrations were much lower, closer to values at the same depths at stations not strongly affected by the eruption.

### Picophytoplankton and bacterioplankton abundances

**Figure 4:**
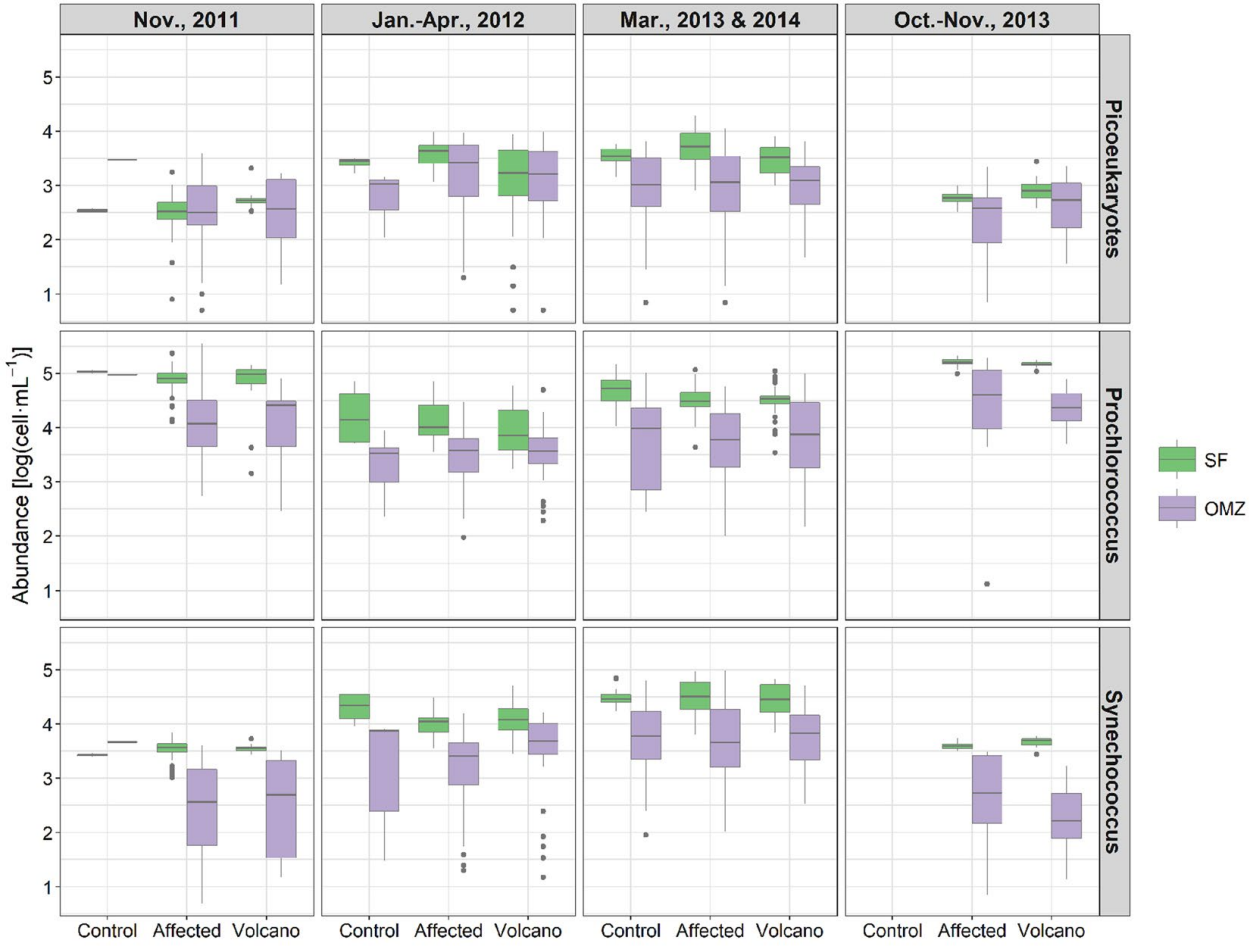
Picophytoplankton abundances (log(cell·mL^−1^)) grouped by sampling period, location and depth. From top to bottom: picoeukaryotes, *Prochlorococcus* and *Synechococcus*. Periods: Nov., 2011 (cruises 1 and 2), Jan.–Apr., 2012 (cruises 3‒7), Mar., 2013 and 2014 (cruises 8 and 10), Oct.–Nov., 2013 (cruise 9). Locations: control (control stations), volcano (stations near the volcano) and affected (any other station affected by the eruption). Depths: SF (subsurface waters, 0–70 m) and OMZ (oxygen minimum zone waters, 70–200 m). In the boxes, the lower and upper hinges correspond to the 25th and 75th percentiles. The upper (lower) whisker extends from the hinge to the largest (smallest) value no further than 1.5 * IQR from the hinge, where IQR is the inter-quartile range, i.e., the distance between the 25th and 75th percentiles. Data beyond the end of the whiskers are plotted individually.

*Synechococcus sp*. (Syn) and *Prochlorococcus sp*. (Proc) type cyanobacteria, as well as picoeukaryotes (Peuk) ‒the most abundant size-fractions of phytoplankton and responsible for most of the Chl-a in the subtropical NE Atlantic (see discussion)‒ were enumerated to see their temporal evolution along the study (Fig. 4). Peuk and Syn followed a similar pattern, with lower average abundances during autumn (the less productive period; cruises 1, 2 and 9) and higher ones during the more productive late winter/spring period (cruises 3‒7, 8 and 10). On the contrary, Proc was more abundant during the autumn cruises.

These seasonal differences were significant (p < 0.0001) for all the three groups in the SF zone, but only occasionally for the OMZ. Differences in abundances between samples from the SF and the OMZ within each cruise were also significant (p < 0.0001) for the three groups. However, there were not significant differences between clustered sampling sites (control/affected/volcano) with any of the picophytoplankton groups at any depth zone during a same cruise.

Phytoplankton biomasses (Supplementary Fig. S2) followed the same pattern of distribution than the abundances, with dominance of Proc during the autumn period and co-dominance of Syn and Peuk during late winter/spring. The fact that Peuk did not dominate in biomass at any period, and that larger phytoplankton were almost absent in the cytometric analyses, indicate that picophytoplankton was the dominant size fraction in the phytoplankton community.

**Figure 5:**
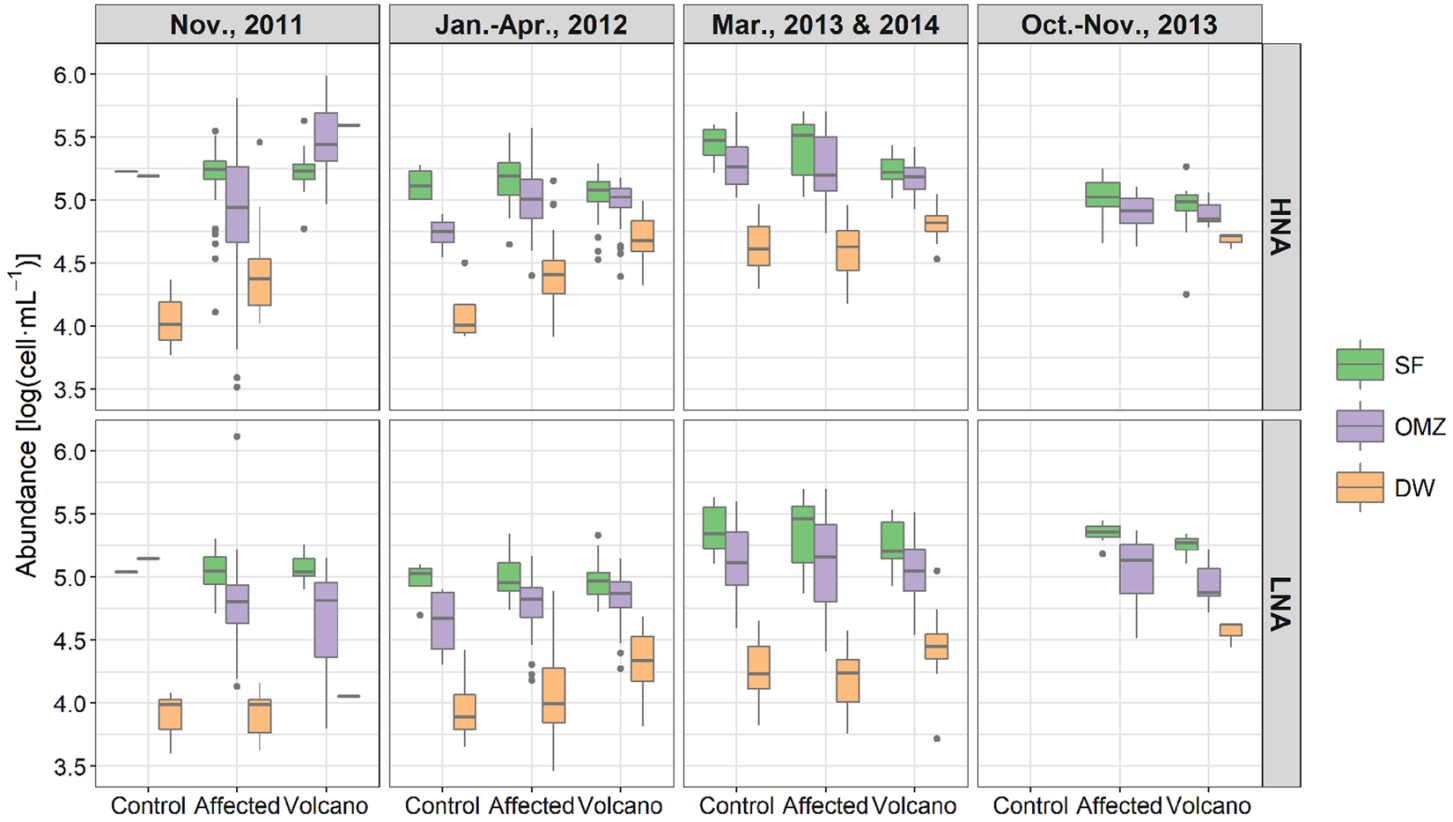
Bacterioplankton abundances (log(cell·mL^−1^)) grouped by sampling period, location and depth. From top to bottom: high nucleic acid (HNA)-content and low nucleic acid (LNA)-content bacteria. Periods: Nov., 2011 (cruises 1 and 2), Jan.–Apr., 2012 (cruises 3‒7), Mar., 2013 and 2014 (cruises 8 and 10), Oct.–Nov., 2013 (cruise 9). Locations: control (control stations), volcano (stations near the volcano) and affected (any other station affected by the eruption). Depths: SF (subsurface waters, 0–70 m) and OMZ (oxygen minimum zone waters, 70–200 m). In the boxes, the lower and upper hinges correspond to the 25th and 75th percentiles. The upper (lower) whisker extends from the hinge to the largest (smallest) value no further than 1.5 * IQR from the hinge, where IQR is the inter-quartile range, i.e., the distance between the 25th and 75th percentiles. Data beyond the end of the whiskers are plotted individually. Bacterioplankton data from cruises 1‒7 corresponds to previously published data^8^, which has been analysed in conjunction with cruises 8‒10.

On the contrary to phytoplankton, the bacterioplankton community showed a clear response to the eruption. Bacterial abundances from cruises 1‒7 (previously published data^8^) show that large and active bacteria (the HNA group) significantly increased (p < 0.001) in OMZ waters and deep waters (DW) above the volcano with respect to other surrounding stations, during the peak of the eruptive phase (Nov., 2011; Fig. 5 upper panels). In contrast, SF maintained similar HNA levels in all three zones. During cruises 3–7 (Jan.‒Apr., 2012) there was a significant decrease (p < 0.0001) in HNA cell abundance in OMZ waters over the volcano, presenting similar levels to those at the affected and control stations. In any case, there was still significantly higher HNA abundances in DW at the affected and, especially, volcano stations with respect to the control. Unlike HNA bacteria, LNA bacteria (Fig. 5, lower panels), did not show significant differences between sampling sites at any of the depth zones. The percentage of HNA bacteria with respect to the total (Supplementary Fig. S3) was always higher in deep waters, as expected, but increased significantly (p < 0.0001) with depth during the first stages of the eruption.

### Remote sensing Chl-a

**Figure 6:**
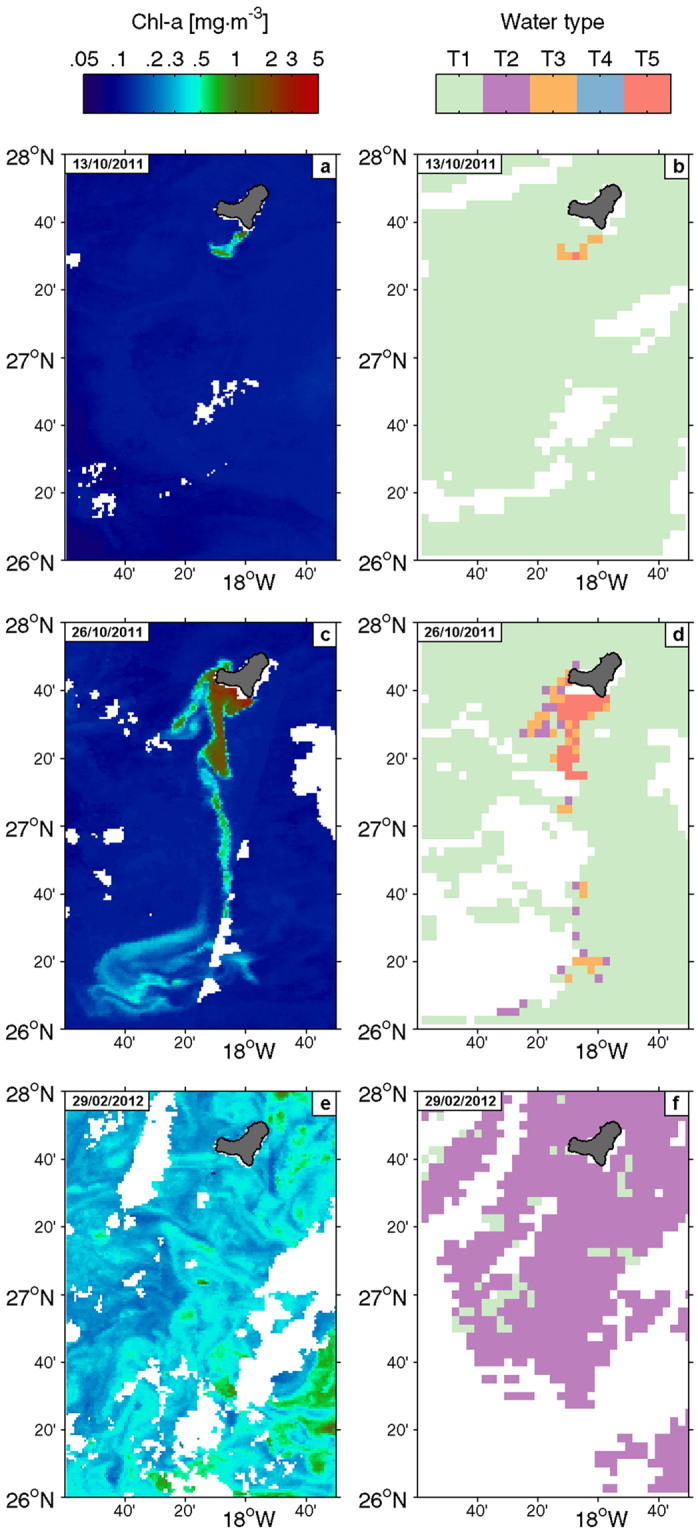
Remote sensing Chl-a (left column, mg·m^−3^) derived from ocean colour and corresponding water classification (right column) for selected days: (**a** and **b**), 13/10/2011; (**c** and **d**), 26/10/2011; (**e** and **f**), 29/02/2012. Water classification scheme: T1: clear waters; T2: moderate, Chl-a-dominated waters; T3: moderate, not Chl-a-dominated waters; T4: turbid, Chl-a-dominated waters; T5: turbid, not Chl-a-dominated waters (see text for details). Maps were generated using Matlab 7.14 R2012a (https://www.mathworks.com/products/matlab).

The volcanic material emission plume was clearly distinguishable by remote sensing because it interfered with the Chl-a ocean-colour signal (Fig. 6a,c,e). During the first months of the eruption, the plume was consistently detected south of El Hierro, being transported southward by mesoscale eddies. Satellite Chl-a values within the plume signal exceeded 1–2 mg·m^−3^. These concentrations were however unrealistic. As an example, *in situ* Chl-a measurements in the upper 25 m of an eddy spun off from south of El Hierro ranged from 0.11 to 0.18 mg·m^−3^ (Supplementary Fig. S4) in opposition to concentrations above 0.5 mg·m^−3^ reported by remote sensing data. After nearly two months of eruption, the plume signal started to weaken, although values were still high over the volcano during Dec., 2011 and Jan., 2012, completely disappearing by Feb., 2012 (Fig. 6e). Results from the combined analysis (see *Data and Methods*) of the diffuse attenuation coefficient for downwelling irradiance at 490 nm ‒K_d_(490), an apparent optical property that governs the propagation of light through water‒ and remote-sensing reflectance ‒R_rs_(λ), water-leaving radiance normalized by the downward irradiance just above the surface at a certain wavelength λ‒ further confirmed that the reported Chl-a signal of the plume was unrealistic. Waters affected by the plume were classified as not Chl-a-dominated (Fig. 6b,d), contrasting with the moderate Chl-a-dominated waters (with values around 0.3 mg·m^−3^), identified during late Feb., 2012 (Fig. 6f), which matched the *in situ* measurements of ~0.2–0.35 mg·m^−3^ during cruise 5.

**Figure 7:**
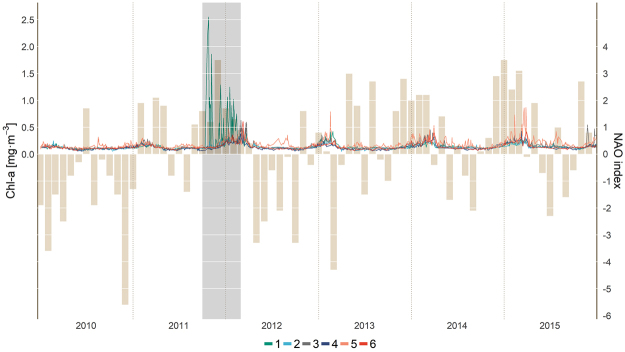
Time-series of remote sensing Chl-a (mg·m^−3^) derived from ocean colour (lines) for selected points in the archipelago (see Fig. 1a) and monthly station-based North Atlantic Oscillation (NAO) index time-series (bars). Source: NCAR (https://climatedataguide.ucar.edu/climate-data). The shaded area indicates the eruptive phase of the volcano (Oct., 2011–Feb., 2012).

Derived ocean colour Chl-a time-series (Fig. 7) placed in context the entire process, showing the high anomalous values of Chl-a at station 1 (the volcano; Fig. 1a) during the eruptive phase (Oct., 2011–Feb., 2012). During this period, reported remote sensing Chl-a estimates were higher than the corresponding *in situ* measurements within plume waters (up to more than ten-fold, see Supplementary Fig. S5 for a match-up). Once the eruption ceased and the emission plume disappeared, Chl-a reached values similar to measured *in situ* concentrations. The end of the eruptive episode (Feb., 2012) coincided in time with the height of the late winter Chl-a maximum. It is worth noting that the winter of 2012 presented relatively high Chl-a values compared to other years, presumably advected from the NW Africa coastal upwelling region (Fig. 7; Supplementary Fig. S6), as discussed below.

## Discussion

Potential ocean fertilization caused by aeolian deposition of volcanic ash has drawn interest over recent years^10–12^. Early works using remote sensing data, such as the ones carried out following the eruptions of Miyake-jima (Japan)^13^ and Soufrière Hills (Montserrat)^14^ volcanos, already suggested a fertilization effect by volcanic ash through the addition of ammonium sulfate and Fe. Strong evidence of volcanic Fe fertilization of the surface ocean was reported around the Aleutian Islands, from MODIS Chl-a data, after the eruption of the Kasatochi volcano, in August 2008^15^. The eruption led to a massive fertilization of the surface waters of the iron-limited north-east Pacific with thousands of tons of Fe, triggering the development of an extensive phytoplankton bloom that increased the surface chlorophyll concentration by 150%. Posterior *in situ* observations confirmed the presence of this bloom that persisted for two months, until October 2008^16,17^. However, clear and marked responses, as the one associated with the Kasatochi eruption, do not always occur^18^. For instance, the Eyjafjallajökull volcano eruption (Iceland, April 2010) was deemed to have had a modest fertilization effect. Although a moderate response in remote sensing Chl-a was detected^19^, the phytoplankton enhancement was short-lived and the prolonged bloom that was observed during the following summer and autumn was suggested to be related to an extremely negative NAO index anomaly that altered the hydrological conditions in the Irminger basin^20^. Thus, there are still uncertainties on the capacity of volcanic ash to act as a fertilizer, pointing to the need to improve our knowledge on the processes and impacts associated with volcanic fertilization of the surface ocean. Several factors, including the amount of ash expelled to the atmosphere, the nutrient content of the ash and its bioavailability (observed values for Fe are usually ~7.5% by weight and <0.1% of total, respectively^19^), nutrient colimitation^21^ and macro/micronutrient limitation patterns in the world ocean^22^, need to be considered.

Unfortunately, if there are few studies on surface ocean impacts caused by the deposition of aerial volcanic ashes, there are almost no observations on the effects of shallow submarine eruptions on the pelagic biota. Very few eruptions have been studied in this regard^23^, the most notorious one probably been the submarine eruption of the Home Reef volcano (Tonga, south west Pacific Ocean), which quickly evolved into a subaerial event upon the formation of an ephemeral island^24^. By combining different data obtained from remote sensors (including MODIS Chl-a data), a plume of discoloured water with a length of 350 km and a diameter of 120 km was reported. The plume was identified as a potential phytoplankton bloom, presumably dominated by the colonial cyanobacteria *Trichodesmium sp*., contributing to Chl-a values 17 times higher than the background sea^24^; although it must be indicated that no *in situ* validation was carried out.

For this reason, the eruption of the shallow submarine volcano south of El Hierro Island, in October 2011^3^, provided a unique opportunity to study the impacts on the almost pristine pelagic community of the marine reserve south of the island, where the volcano is placed. The strong physical and chemical perturbations that occurred in the water column after the eruption (particularly related to oxygen depletion) resulted in the reduction of epipelagic fish and zooplankton stocks and a disruption of the diel vertical migration of mesopelagic organisms^7^. However, once the eruption ceased, the distribution and abundance of the pelagic fauna returned to pre-eruption levels. Associated with this event, high concentrations of Fe(II), phosphate, nitrate and silicate were reported in the water column above the volcano during its eruptive phase (October, 2011 to February, 2012)^5^. Nutrient peaks were however restricted to low (almost anoxic) and narrow (about 50–75 m thick) oxygen minimum zones, resulting from the oxidation of reduced compounds expelled by the volcano and accumulated at the upper pycnocline^3^. Based on these data, it was suggested that the same eruption that caused a damaging effect on the marine biota would have produced a fertilization, yielding the necessary nutrients to rapidly restore the ecosystem south of the island^5^. However, no information was provided on the spatial distribution of these nutrients beyond the station situated over the volcano, necessary to account for the potential fertilization of the marine biota south of the Island. Moreover, in a more recent study^4^ –where the observations were extended with three more cruises carried out in October 2013, March 2014 and May 2015‒ it was shown that total Fe (II) concentrations were high in the water column above the volcano (mostly below 100 m) but dropped to values < 0.5 nM a few miles away the cone.

With the aim of testing the biological fertilization hypothesis, we analysed the temporal and spatial variability of Chl-a and picophytoplankton during and after the eruptive phase. We also placed in context the observed changes in Chl-a south of El Hierro with respect to the Chl-a evolution in other regions of the Canary Islands waters. The Chl-a and phytoplankton results do not show any bloom related to the volcano eruption, neither during nor after the eruptive phase. Although multiple patches of abnormally high remote sensing Chl-a were initially observed when the most intense eruptive episodes occurred, these were not associated with phytoplankton blooms. Rather, they were erroneous estimates consequence of the interference produced by discoloured waters that contained great amounts of sulphur compounds^25^. These produced a signal that interfered with that of Chl-a, resulting in unrealistic estimates by the remote sensing Chl-a algorithms, as confirmed when comparing with *in situ* Chl-a measurements. Our water type classification analysis, based on a previous study carried out in the same area^25^, shows that plume waters were classified as not Chl-a-dominated (Fig. 6). Although occasionally some pixels classified as Chl-a-dominated waters appeared in the border of the plume, they were misclassified probably due to the dilution of the plume materials. The discoloured southward-drifting plume showed much lower *in situ* Chl-a concentrations than remote sensing estimates (Supplementary Fig. S4), giving no evidence of downstream fertilization. The seasonal variability in the magnitude of Chl-a data measured from samples collected *in situ*, agreed with previous studies of Chl-a on the Canary region^26^. Moreover, the highest Chl-a values (up to 0.5 mg·m^−3^) observed at a shallow (<250 m) station (St. 5/15) south of El Hierro are in the range of published studies in coastal waters of the islands. For instance, Chl-a concentrations up to 1 mg·m^−3^ during winter blooms in coastal waters of Gran Canaria Island have been previously reported^27,28^.

The effect of the volcanic plume in the Chl-a time-series was clearly visible (Fig. 7). Multiple peaks of extremely high satellite Chl-a concentrations (1‒2.5 mg·m^−3^ vs typical *in situ* values < 0.2 mg·m^−3^) were observed south of El Hierro and occasionally north (Fig. 7, points 1 and 2, respectively), between Oct., 2011 and Feb., 2012. Again, these values were unrealistic and did not indicate the development of any algal bloom. After the eruptive episode (by Feb., 2012), satellite Chl-a at El Hierro (Fig. 7, point 1) was restored to average values of other ocean regions of the archipelago. Although seasonal and interannual variability in Chl-a were low during the 6 years (2010–2015) of our time series analysis (Fig. 7), the small changes were caused by natural variability and occurred synchronically at all the study points. Relatively higher Chl-a was observed during the late winter bloom, when the seasonal thermocline weakens as the result of surface cooling, enhancing the diffusion of nutrients from below to the euphotic zone^27^. There was however some interannual variability in Chl-a between different winters, which seemed to be caused by the export of Chl-a from the NW Africa coastal upwelling to the Canary region (Supplementary Fig. S6). The intensity of the export varied from year to year and appeared to be positively correlated with the North Atlantic Oscillation (NAO). Indeed, winters with higher Chl-a (e.g., 2012, 2014 and 2015) were generally preceded by months of positive NAO indices, whereas winters with lower Chl-a (e.g., 2010 and 2011) were associated with negative NAO indices (Fig. 7). Upwelling favourable winds have been described to be positively correlated to the NAO index^29,30^ and thus they are deemed a potential link that could explain the relation.

The phytoplankton community in our study was dominated by small picoplankton, as it is expected from the low Chl-a values recorded, and has been described in the past^31,32^. Proc, Syn and Peuk abundances were in the same ranges as those reported by other authors for the eastern subtropical North Atlantic^33,34^. However, abundances within each group significantly differed depending on the sampling period. Proc showed higher concentrations during cruises carried out in autumn, when waters were warmer and more stratified, while Syn and Peuk were more abundant during the late winter bloom. These changes agree with the distributions observed in the literature^33,35^, where Proc is reported to be dominant in warm, oligotrophic waters, whereas Syn and Peuk predominate in cooler, mesotrophic waters. Changes were significant between most of the cruises in subsurface waters (0‒70 m) for all three groups. Moreover, the fact that no significant changes were found between control, affected and volcano stations suggests a limited impact, if any, on picoplankton group abundances after the eruption.

Bacterioplankton, on the contrary, displayed a clear response during the eruptive phase^8^, showing a clear increase in the percentage of HNA bacteria (the most active cells) after the eruption. However, once the eruption ceased, the HNA abundances were restored to values similar to the control stations. Our data (Fig. 5 and Supplementary Fig. S3) extends previously published results^8^ with three more cruises (8–10), without varying their conclusions.

In summary, the impact of the eruption of the El Hierro volcano on the phytoplankton community was not evident, and thus the assertion that the input of nutrients by the volcano provided the grounds for a fast recovery of the ecosystem in the marine reserve south of El Hierro lacks evidential weight. On the contrary, our results show that temporal changes in chlorophyll and phytoplankton abundance and biomass were caused by natural variability rather than by any nutrient fertilization produced by the eruption. It could be both that nutrient fertilization was a very local phenomena restricted to the water column above the volcano, and that an efficient renewal of waters in the zone diluted the nutrients as to have a visible impact on phytoplankton growth south of the island. The strong southward drift of the discoloured plume of water, caused by the expelled material, would give support to this hypothesis.

## Data and Methods

### Sampling

The samples were collected along 10 oceanographic cruises (Fig. 1c) carried out in the frame of three research projects–Bimbache (BBC), Guayota (GYT) and Vulcano (VUL)–during both the eruptive (10/2011–02/2012) and post-eruptive (from 03/2012 on) phases of the volcanic episode. The first samples were collected after 3 weeks from the onset of the eruption in cruise 1 (BBC 3, 4–9 Nov., 2011) coinciding with the strongest bubbling episode, followed by cruises 2 (BBC 5; 16–20 Nov., 2011), 3 (BBC 8; 13–15 Jan., 2012), 4 (BBC 10; 9–12 Feb., 2012), 5 (BBC 12 12; 24–26 Feb., 2012), 6 (GYT 2; 17 Mar., 2012), 7 (GYT 3; 28 Apr., 2012), 8 (VUL 1; 22 Mar., – 5 Apr., 2013), 9 (VUL 2; 26 Oct., – 11 Nov., 2013) and 10 (VUL 3; 4–24 Mar., 2014).

### *In situ* Chl-a and CTD-fluorescence

*In situ* Chl-a was estimated by means of a Turner Designs bench fluorometer previously calibrated with pure chlorophyll *a*. Water samples were collected in 0.5 L bottles, filtered using Whatman GF/F filters and preserved at −20 °C until analysis. Pigments were extracted in cold acetone (4 °C) for 24 h. For the final determination of Chl-a, the acetone extracts were acidified allowing Chl-a and phaeopigments to be independently estimated.

Fluorescence profiles in the water column were obtained through a Seapoint fluorometer. Fluorescence values corresponding to Niskin bottles were paired with *in situ* Chl-a measurements and linear fits were calculated (r^2^ > 0.75) in order to calibrate the fluorometer and to derive Chl-a profiles.

### Picophytoplankton and bacteria

Heterotrophic bacteria, small photosynthetic eukaryotic cells (picoeukaryotes), and *Prochlorococcus* sp. and *Synechococcus* sp. type cyanobacteria, were enumerated with a FACScalibur (Becton and Dickinson) flow cytometer. Samples (4 mL) were fixed with a 2% final concentration of paraformaldehyde, incubated for 30 min at 4 °C and then stored frozen in liquid nitrogen until analysis. To count bacteria, samples were thawed and 400 μl were stained with DMS-diluted SYTO-13 (Molecular Probes Inc.) stock (10:1) at 2.4 μM final concentration. Bacteria were identified by their signatures in a plot of side scatter (SSC) versus green fluorescence (FL1). High nucleic-acid content (HNA) bacteria and low nucleic-acid content (LNA) bacteria were separated in the scatter plot. Cyanobacteria were subtracted from HNA bacteria estimates when they overlapped. The bacterioplankton analysis includes previously published^8^ data (cruises 1‒7) along with unpublished one (cruises 8‒10). Samples were run at low speed for bacteria (18 μL min^−1^) and medium speed for the other photosynthetic cells (70 μL min^−1^). A suspension of yellow–green 1 µm latex beads (∼10^6^ beads ml^−1^) was added as an internal standard (Polysciences, Inc.).

Biomasses of *Prochlorococcus*, *Synechococcus* and picoeukaryotes were estimated by multiplying the abundance data obtained by flow cytometry by the average cell carbon content calculated from each group, using the conversion factors obtained by Montero *et al*. (unpublished) from samples collected in coastal waters of Gran Canaria: 50 fg C·cell^−^1 for *Prochlorococcus*, 100 fg C·cell^−1^ for *Synechococcus* and 444 fg C·cell^−1^ for picoeukaryotes.

### Remote sensing Chl-a

Chl-a data at 1 km^2^ spatial, and daily temporal resolutions was downloaded from the Copernicus Marine Environment Monitoring Service (CMEMS) website (marine.copernicus.eu). The employed product (ref. OCEANCOLOUR_ATL_CHL_L3_REP_OBSERVATIONS_009_067) has been developed by the Plymouth Marine Laboratory’s (PML) Remote Sensing Group and combines Moderate-resolution Imaging Spectroradiometer (MODIS) and Visible Infrared Imaging Radiometer Suite (VIIRS) data. Chl-a time-series were constructed for selected points in the archipelago (Fig. 1a): one above the volcano area, and five north and south of El Hierro, Tenerife and Gran Canaria islands, which were intended to represent background values for upstream/downstream conditions. However, materials expelled by the volcano, which contained abundant sulphur species, discoloured sea water and interfered in remote sensing measurements, resulting in unrealistic Chl-a concentrations. In order to avoid this issue, remote sensing Chl-a data was analysed using a method previously applied in this same area^25^. Briefly, the diffuse attenuation coefficient for downwelling irradiance at 490 nm (K_d_(490), an apparent optical property that governs the propagation of light through water^36^) was employed to classify waters depending on their turbidity and identify areas affected by the plume:

1. Clear waters: K_d_(490) < 0.05.
2. Moderate waters: 0.13 > K_d_(490) ≥ 0.05.
3. Turbid waters: K_d_(490) ≥ 0.13.

Both turbid and moderate waters were further divided into two subgroups based on the ratio of remote-sensing reflectance (R_rs_ (λ), water-leaving radiance normalized by the downward irradiance just above the surface at a certain wavelength λ^37^) for 667 nm and 678 nm bands:

$$\frac{{{\rm{R}}}_{{\rm{rs}}}(667)}{{{\rm{R}}}_{{\rm{rs}}}(678)}$$

Considering the maximum absorption peak of Chl-a at 665 nm, waters with a ratio value below 1.0 were classified as Chl-a-dominated, whereas those presenting a ratio above 1.0 were regarded as not Chl-a-dominated. According to this, waters were divided into five types: clear waters (T1); moderate, Chl-a-dominated waters (T2); moderate, not Chl-a-dominated waters (T3); turbid, Chl-a-dominated waters (T4); turbid, not Chl-a-dominated waters (T5). K_d_(490) and R_rs_ data were downloaded from NASA’s Ocean Color portal (oceancolor.gsfc.nasa.gov) at 4 km^2^ spatial, and daily temporal resolutions.

### Statistical analysis

Following a sample classification scheme previously employed in this same volcanic event^8^, Chl-a and flow cytometry samples were grouped according to time of sampling, sampling site and sampling depth. Oceanographic cruises were clustered into four distinct sampling periods: cruises 1‒2 (Nov., 2011), 3‒7 (Jan.–Apr., 2012), 8 and 10 (Mar., 2013 and 2014, respectively), and 9 (Oct.–Nov., 2013). Similarly, samples were separated into three groups depending on which area they were collected: control, affected and volcano (Fig. 1b, Supplementary Table S1). The *volcano* stations were those situated just above the volcano or in its close vicinity and, thus, were subject to direct and constant influence from the eruption. The *affected* stations, although not been immediately close to the volcano, were influenced by the eruption due to the spreading of the volcanic plume ‒this influence being presumably weaker than in the *volcano* stations and also variable as a consequence of the variation in the spreading pattern of the plume due to mesoscale activity. The *control* stations were located in far-field areas believed to be free of any interaction with the eruption. However, this was not always true and, eventually, when these stations were under the effect of the volcanic plume, they were clustered under the *affected* group (see Supplementary Table S1 for station classification details). Samples were also classified according to their depth, based in part on dissolved oxygen distribution, published elsewhere^3,5,8^: surface and subsurface (SF) waters: 0–70 m; oxygen minimum zone (OMZ) waters: 70–200 m; deep waters (DW): >200 m.

To assess whether significant differences were present between the different groups, ANOVA accompanied by *post hoc* Tukey-Kramer tests were carried out when normalization of data was achieved. Alternatively, for data which did not follow a normal distribution Kruskal-Wallis tests supported with *post hoc* Conover tests were performed. For all tests a significance level of 0.05 was considered. Statistical analyses were carried out using R. Plots were also made with R using the *ggplot2* package; except maps, which were done in Matlab (*M_Map* package). CTD-fluorescence data analyses were preformed making use of the *oce* package for R (https://dankelley.github.io/oce/).

### Data availability

The datasets generated during and/or analysed during the current study are available from the corresponding author on request.

## Electronic supplementary material

Supplementary Information
