# Supplementary material for: Lack of impact of the El Hierro (Canary Islands) submarine volcanic eruption on the local phytoplankton community
Source: Sci Rep. 2018 Mar 16;8:4667. doi: 10.1038/s41598-018-22967-6 (PMC5856795; doi:10.1038/s41598-018-22967-6)
Supplement: Supplementary file 1 — Supplementary Information [file 41598_2018_22967_MOESM1_ESM.pdf]

Lack of impact of the El Hierro (Canary Islands) submarine volcanic eruption on the local phytoplankton community

M. Gómez-Letona[1,*], J. Arístegui[1], A.G. Ramos[2], M.F. Montero[1], J.Coca[2],

[1] Instituto de Oceanografía y Cambio Global (IOCAG), Universidad de Las Palmas de Gran Canaria, ULPGC, Las Palmas de Gran Canaria, Spain.

[2] División de Robótica y Oceanografía Computacional, IUSIANI, Universidad de Las Palmas de Gran Canaria, ULPGC, Las Palmas de Gran Canaria, Spain.

* Corresponding author: markel.gomez101@alu.ulpgc.es

Supplementary Table S1

Supplementary Figures S2 – S6

Station metadata

Supplementary Table S1. Metadata of samples for *in situ* Chl-a and flow cytometry analyses. Stations marked with an *R* correspond to those repeated over several cruises; for those stations, the code (SSRN) should be read as: SS, station number; R, “repetition”; and N, number of the repetition. For instance, “03R1” would be the 1st repetition of the original station 03. * depths or cruises in which no samples for *in situ* Chl-a measurements were collected. ⁑ depths in which no samples for the flow cytometry analysis were collected.

Cruise	Station	Longitude	Latitude	Depths [m]					Location
	01	-17.914833	27.655	5	25*	75			Control
	03	-17.993	27.618	5*					Volcano
	04	-18.006333	27.6291667	25	50	75	150	266*	Volcano
	05	-18.029	27.6588333	5	25	75	100	125	Affected
	06	-18.066667	27.6551667	5	25	75			Affected
	08	-18.216	27.7305	5	25	50	75		Affected
	09	-18.214833	27.6928333	5⁑	25⁑	50⁑	75⁑	150⁑	Affected
	10	-18.1405	27.655	25	50	62*	150		Affected
	11	-18.140333	27.621	5	25	63	76	150	Affected
BBC3 (4−9 Nov., 2011)	12	-18.066667	27.5811667	5	25	50	70	150	Affected
	14	-17.990167	27.544	5	25	50	75	150	Affected
	15	-17.988667	27.581	5	25*	50	64	150	Affected
	17	-18.0625	27.5431667	5	25	50	70	150*	Affected
	18	-18.2145	27.655	5	25	50	83	105	Affected
	20	-18.141167	27.5461667	5	25	50	78	167	Affected
	22	-17.989167	27.6253333	20*	75*	90*			Volcano
	23	-17.9945	27.6246667	10	75	90	190*		Volcano
	24	-18.0075	27.6293333	5	25	50	75		Volcano
	914	-18.486833	27.057	5	25	50	75	150	Affected
BBC5 (16−20 Nov., 2011)	03R1	-17.995167	27.618	5	25	50	75	160*	Volcano

	03R2	-17.995167	27.6178333	5	25	50	75	100	125	Volcano
	04R1	-18.006167	27.6295	5	25	50	75	125		Volcano
	04R2	-18.006333	27.6283333	5	25	50				Volcano
	05R1	-18.03	27.6591667	5	25	50	75	125		Affected
	05R2	-18.031	27.6593333	5	25	50	75	100	125	Affected
	01	-18.185	27.7595	5	25	50	75	150		Affected
	02	-18.223333	27.7786667	5	25	58	75	100		Affected
	03	-18.203667	27.8201667	5	25	50	75	100		Affected
	04	-18.171167	27.795	5⁑	75⁑	100⁑				Affected
	05	-18.109167	27.773	5	25	55	75	100		Affected
	06	-18.125667	27.7986667	5	25	50	75	100		Affected
	07	-18.164667	27.8228333	5	25	50	88	105		Affected
	08	-18.117167	27.8243333	5	25	50	80	100		Affected
	09	-18.092	27.7986667	5	25	50	75	100		Affected
	10	-18.062667	27.7886667	5	25	50	75	100		Affected
	11	-18.0395	27.7791667	5	25	50	75	100		Affected
	12	-18.043833	27.8208333	5	25	50	75	100		Affected
	13	-18.077333	27.8271667	5	25	50	75	100		Affected
	14	-18.096333	27.853	5	25	50	75	100		Affected
	15	-18.039333	27.8823333	5	25	50	75	100		Affected
	16	-18.034667	27.8511667	5	25	50	75	100		Affected
	17	-18.012333	27.8263333	5	25	50	75	100		Affected
	18	-17.9845	27.8498333	5	25	50	75	100		Affected
	19	-17.991167	27.8776667	5	25	50	73	100		Affected
BBC8 (13−15 Jan., 2012) *	01	-17.895	27.7695	5	44	50	75	100		Affected
	02	-17.914833	27.7341667	5	25	75	100	150		Affected

	03	-17.957833	27.656	5	50	75	100	150				Affected
	04	-17.956667	27.6191667	5	50	75	100	150				Affected
	05	-17.958667	27.683	5	50	75	100	150	200			Affected
	06	-17.917167	27.6803333	5	50	75	100	150				Affected
	07	-17.917667	27.7073333	5	50	85	100	150				Affected
	08	-17.947167	27.7058333	5	50	75	100	150				Affected
	09	-17.881167	27.7485	5	50	75	100	150				Affected
	01R1	-17.915	27.6556667	5	50	75	100	150				Control
	02R1	-17.9145	27.6183333	5	25	75	100	150				Affected
	03R3	-17.989167	27.6183333	5	25	50	100					Volcano
	04R3	-18.005667	27.6285	5	50	75	86	150				Volcano
	05R3	-18.028167	27.659	5	50	75	100	150				Affected
	21R1	-17.997333	27.6105	25	50	75	100	150				Volcano
	23R1	-17.994667	27.6246667	5	57	75	100	150				Volcano
BBC10 (9−12 Feb., 2012)	01	-18.0675	27.6843333	5	25	50	75	100	150	200*	400*	Affected
	02	-18.066667	27.6576667	5	50	150	200*					Affected
	03	-18.066667	27.6293333	5*	25*	50	75*	100*	150*	200*		Affected
	04	-18.0675	27.612	5	50	150	200*					Affected
	05	-18.064333	27.5908333	5	50	150	200*					Affected
	06	-18.027833	27.6283333	5	50	165	200*					Affected
	07	-18.0265	27.6096667	5	25*	100	200					Affected
	08	-18.025667	27.5851667	5	56	150	200					Affected
	09	-17.996667	27.588	5⁑	50⁑	100⁑	200⁑					Affected
	01R2	-17.914667	27.6621667	5⁑	50⁑	100⁑						Affected
	03R4	-17.996	27.6201667	5⁑	25⁑	50⁑	75⁑	100⁑	150⁑			Volcano
	03R5	-17.9955	27.6186667	5	25	50	100	125				Volcano

	03R6	-17.9955	27.62	5	25	50	100	160						Volcano
	04R4	-18.0055	27.6283333	5	50	100	150	200*						Volcano
	05R4	-18.028833	27.6578333	5	25	50	100	150						Affected
	21R2	-17.998167	27.6083333	5⁑	25⁑	50⁑	75⁑	100⁑	150⁑					Volcano
	21R3	-17.9965	27.6111667	5*	25*	50*	75*	100*	150*					Affected
	23R2	-17.995167	27.625	5	25	50	100	215						Volcano
BBC12 (24−26 Feb., 2012) *	01	-18.0265	27.6281667	5	50	100	200	300	400					Affected
	03	-17.997833	27.5876667	5	50	100	200							Affected
	04	-18.0265	27.5851667	5	50	100	200							Affected
	06	-18.002833	27.62	5	50	100	200	300	423					Affected
	01R3	-17.9135	27.6571667	5	75	125								Control
	03R7	-17.992667	27.6198333	5	20	30	50	70	86					Volcano
	03R8	-17.993833	27.6213333	5	25	50	75	100	165					Volcano
	03R9	-17.995	27.62	5	25	50	75	100	185					Volcano
	04R5	-18.005333	27.6286667	5	25	40	100	200	300					Volcano
	21R4	-17.997833	27.6101667	5	50	100	200	300	400					Volcano
GYT2 (17 Mar., 2012)	04	-18.00567	27.6285	5	25	60	100	125	250					Volcano
	23	-17.99467	27.62467	5	35	50*	75	100	125					Volcano
GYT3 (28 Apr., 2012)	21	-17.99733	27.6105	5	50	75	100	200	300					Volcano
	23	-17.99467	27.62467	5	50	75	100	150	180					Volcano
	V	-17.98917	27.61833	2	25*	50	75	100	115*					Volcano
VUL1 (22 Mar., – 5 Apr., 2013)	02	-17.837	27.7695	5	50	75	100	150	200*	400*	600*	800*	1000*	Control
	04	-17.906333	27.7328333	5	50	75	100	150	200*	400*	600*	800*	1000*	Control
	06	-17.936833	27.67	5	50	75	100	150	200*	400*	600*	800*	1000*	Control
	07	-17.886667	27.687	5	50	60	100	150	200*	400*	600*	800*	1000*	Control
	08	-17.937	27.6195	5	50	75	100	150	200*	400*	600*	800*	1000*	Control

	11	-17.987	27.4698333	5	50	75	100	150	200*	400*	600*	800*	1000*	Affected
	13	-17.987167	27.5698333	5	50	60	100	150	200*	400*	600*	800*	1000*	Affected
	14	-17.986667	27.6201667	5⁑	50⁑	75⁑	100⁑	150⁑						Volcano
	16	-18.036833	27.6198333	5	50	75	100	150	200*	400*	600*	800*	1000*	Affected
	21	-18.1025	27.6546667	5	50	75	100	150	200*	400*	600*	800*	1000*	Affected
	23	-18.136833	27.6848333	5	50	75	100	150	200*	400*	600*	800*	1000*	Affected
	50	-17.990667	27.6156667	5*	50*	75*	100*	150*	200*	315*	323*	326*		Volcano
	51	-17.991333	27.6168333	5*	50*	75*	100*	150*	239*	246*	250*			Volcano
	52	-17.992	27.6176667	5*	50*	75*	100*	150*	197*	205*	209*			Volcano
	53	-17.992	27.6185	5*	50*	85*	100*	150*	176*	183*				Volcano
	54	-17.992667	27.6188333	5*	50*	75*	100*	148*	153*	162*				Volcano
	55	-17.993	27.6193333	5*	50*	75*	109*	113*						Volcano
	56	-17.993333	27.6203333	50*	75*	82*	91*							Volcano
	57	-17.994	27.6203333	5*	50*	75*	100*	115*	123*					Volcano
	58	-17.993167	27.6216667	50*	75*	100*	150*	170*	177*					Volcano
	59	-17.993	27.6221667	5*	50*	75*	100*	158*	163*					Volcano
	5402	-17.992667	27.619	5⁑	50⁑	75⁑	100⁑	162⁑						Volcano
	13	-17.987	27.57	5*	25*	50*	75*	100*	150*					Affected
	15	-18.036833	27.6696667	5*	25*	50*	75*	100*	150*					Affected
	16	-18.0365	27.6198333	5*	25*	50*	75*	100*	150*					Affected
	19	-18.086833	27.6196667	5*	25*	50*	75*	100*	150*					Affected
VUL2 (26 Oct., – 11 Nov., 2013)	20	-18.087	27.6805	5	25	50	75	100	150					Affected
	21	-18.1025	27.6546667	5	25	50*	75	100	150					Affected
	22	-18.137	27.6481667	5	25	50	75	100	150					Affected
	23	-18.136833	27.685	5	25	50	75	100	150					Affected
	50	-17.990667	27.6156667	5*	25*	50*	75*	100*	150*	334*				Volcano

	51	-17.991333	27.6168333	5*	25*	50*	75*	100*	150*	246*	Volcano
	52	-17.992	27.6176667	5	25	50	75	100	150	204*	Volcano
	53	-17.992	27.6185	5*	25*	50*	75*	100*	150*		Volcano
	55	-17.993	27.6193333	5*	25*	50*	75*	100*	150*		Volcano
	56	-17.993333	27.6203333	5*	25*	50*	75*	150*			Volcano
	58	-17.993167	27.6216667	5	25	50*	75	100	150	176*	Volcano
VUL3 (4−24 Mar., 2014)	02	-17.837	27.7695	5	25	50	75	100	150		Control
	07	-17.886667	27.687	25*	65*	75*	150*				Control
	08	-17.937167	27.6195	5	25	50	75	100	150	400	Control
	13	-17.987167	27.5698333	5	25	50	75	100	150		Affected
	15	-18.037	27.6698333	5	25	50	75	100	150		Affected
	16	-18.036833	27.6198333	5*	25*	50*	75*	100*	150*		Affected
	17	-18.036833	27.57	5	25	50	75	100	150	250	Affected
	18	-18.0595	27.6433333	50*	68*	100*	150*				Affected
	19	-18.087167	27.6198333	5	25	50	75	100	150		Affected
	20	-18.087	27.6803333	5	25	50	75	100	150*		Affected
	21	-18.1025	27.6546667	5	25	50	75*	100	150		Affected
	22	-18.137	27.648	5	25	50*	75	100*	150*		Affected
	23	-18.136833	27.6848333	5*	25*	50*	75*	100*	150*		Affected
	50	-17.990667	27.6156667	5	25	50	75	100	150	250*	Volcano
	51	-17.991333	27.6168333	5*	25*	50*	75*	100*	150*	247*	Volcano
	52	-17.992	27.6176667	5	25	50	75	100	150	205*	Volcano
	53	-17.992	27.6185	5*	25*	50*	75*	100*	150*		Volcano
	54	-17.992667	27.6188333	5*	25*	50*	75*	100*	150*		Volcano
	55	-17.993	27.6193333	5	25	75	100	132*			Volcano
	56	-17.993333	27.6203333	5*	25*	50*	75*	92*			Volcano

	58	-17.993167	27.6216667	5*	25	50	75	100	150	Volcano
	61	-17.993167	27.6195	5	25	50	75	100	117	Volcano

Picoplankton biomass

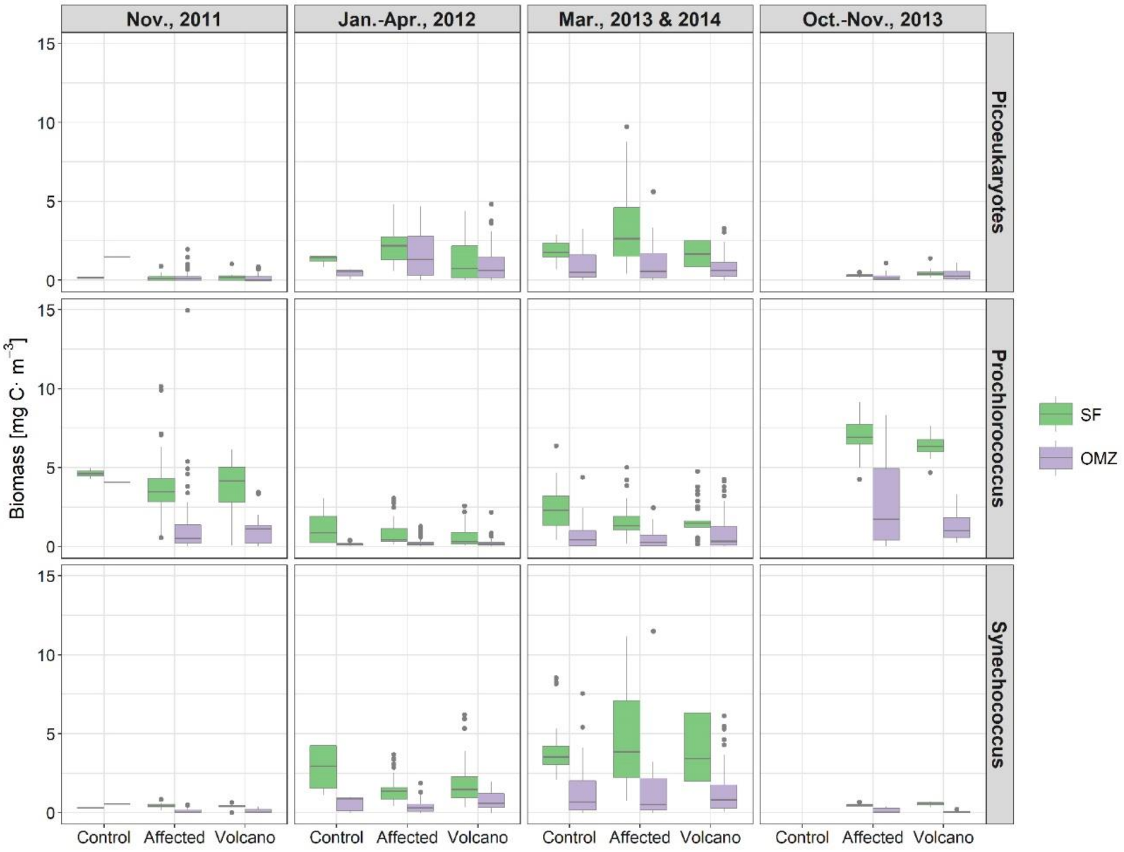

Supplementary Fig. S2. Picoplankton group biomass (mg C·m^{-3}) grouped by sampling period, location and depth. From top to bottom: picoeukaryotes, *Prochlorococcus* and *Synechococcus*. Periods: Nov., 2011 (cruises 1 and 2), Jan.–Apr., 2012 (3–7), Mar., 2013 and 2014 (8 and 10), Oct.–Nov., 2013 (9). Locations: control (control stations), volcano (stations near the volcano) and affected (any other station affected by the eruption). Depths: SF (subsurface waters, 0–70 m) and OMZ (oxygen minimum zone waters, 70–200 m). In the boxes, the lower and upper hinges correspond to the 25th and 75th percentiles. The upper (lower) whisker extends from the hinge to the largest (smallest) value no further than 1.5 * IQR from the hinge, where IQR is the inter-quartile range, i.e., the distance between the 25th and 75th percentiles. Data beyond the end of the whiskers are plotted individually.

HNA %

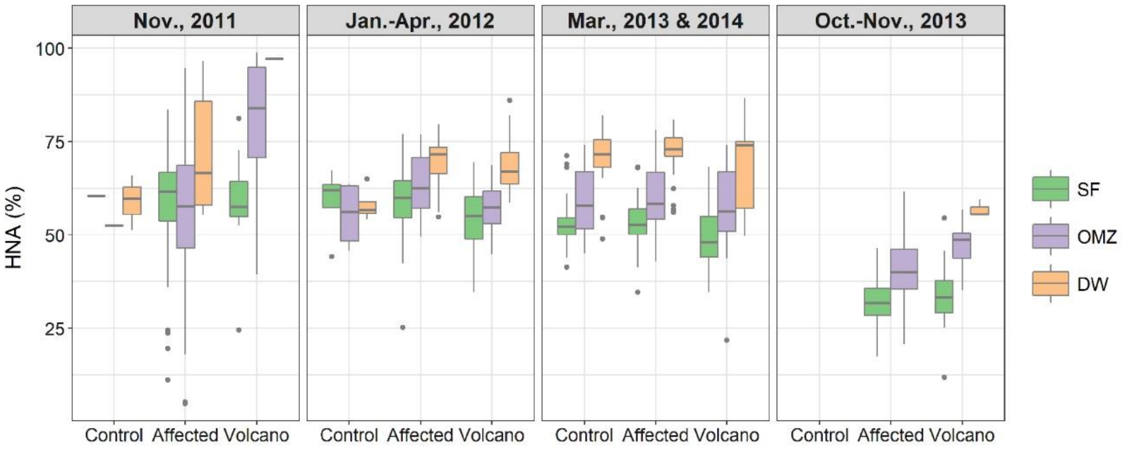

Supplementary Fig. S3. Percentage (%) of high nucleic acid (HNA)-content bacteria grouped by sampling period, location and depth. Periods: Nov., 2011 (cruises 1 and 2), Jan.–Apr., 2012 (3–7), Mar., 2013 and 2014 (8 and 10), Oct.–Nov., 2013 (9). Locations: control (control stations), volcano (stations near the volcano) and affected (any other station affected by the eruption). Depths: SF (subsurface waters, 0–70 m), OMZ (oxygen minimum zone waters, 70–200 m) and DW (deep waters, >200 m). In the boxes, the lower and upper hinges correspond to the 25th and 75th percentiles. The upper (lower) whisker extends from the hinge to the largest (smallest) value no further than 1.5 * IQR from the hinge, where IQR is the inter-quartile range, i.e., the distance between the 25th and 75th percentiles. Data beyond the end of the whiskers are plotted individually. Bacterioplankton data from BBC and GYT cruises corresponds to previously published data[7], which has been analysed in conjunction with VUL cruises.

Chl-a within the southward drifting plume

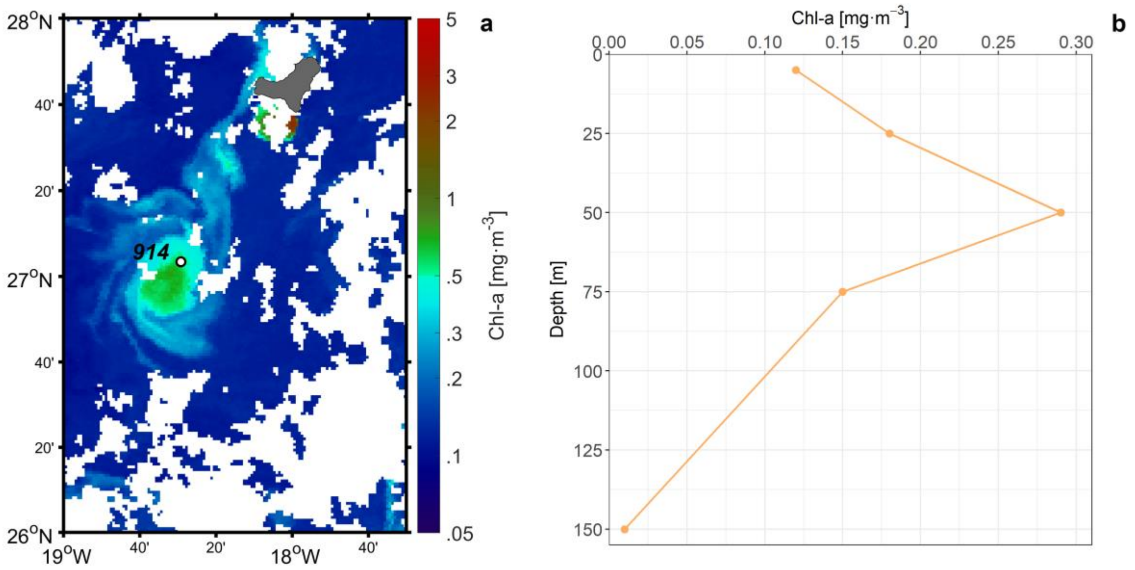

Supplementary Fig. S4. a) Remote sensing Chl-a on 06/11/2011 showing the southward drifting plume along with the position of Station 914 (sampled on 07/11/2011, reportedly still within the plume). b) Concentration of *in situ* Chl-a at station 914.

***In situ* vs remote sensing Chl-a**

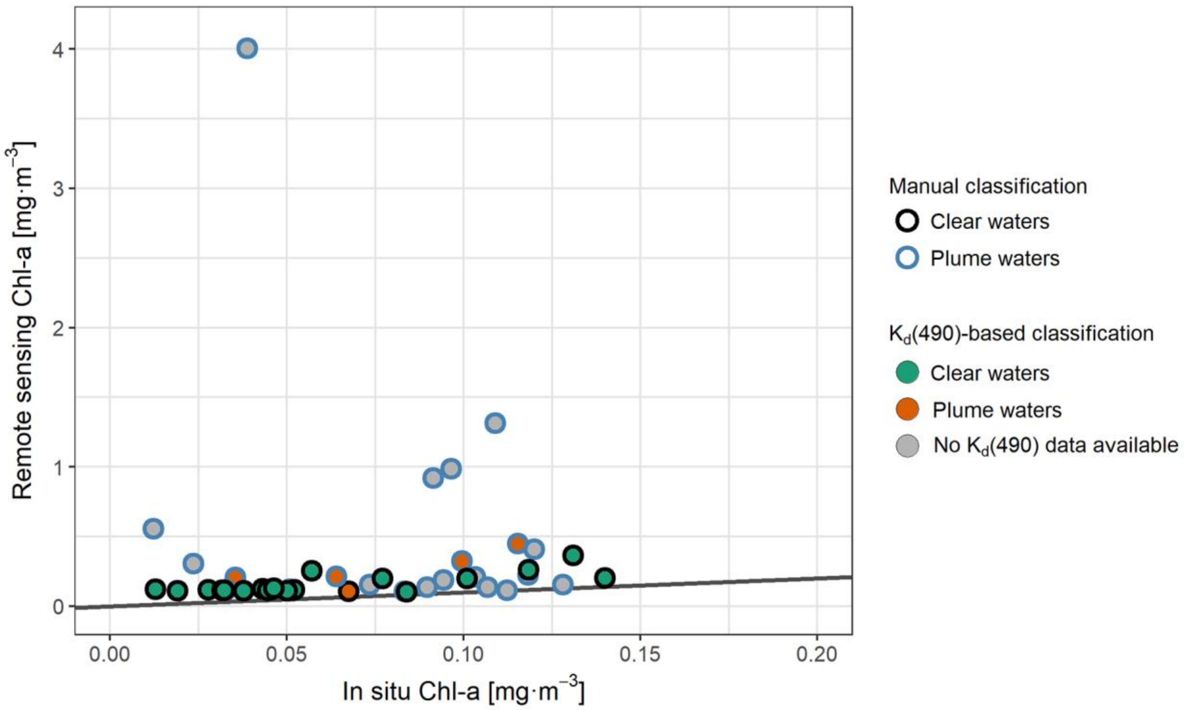

Supplementary Fig. S5. *In situ* and remote sensing Chl-a match-ups. Remote sensing Chl-a values correspond to 3-day-averages of the pixel closest to the sampling station. Waters were classified into clear/plume waters based on K_d(490) values (see *Remote sensing Chl-a* section in *Data and Methods*), and manually. Manual classification was performed assuming that, before Mar., 2012, 'affected' and 'volcano' stations were located within the plume. The grey line represents equal values.

Remote sensing Chl-a in the Canary Islands

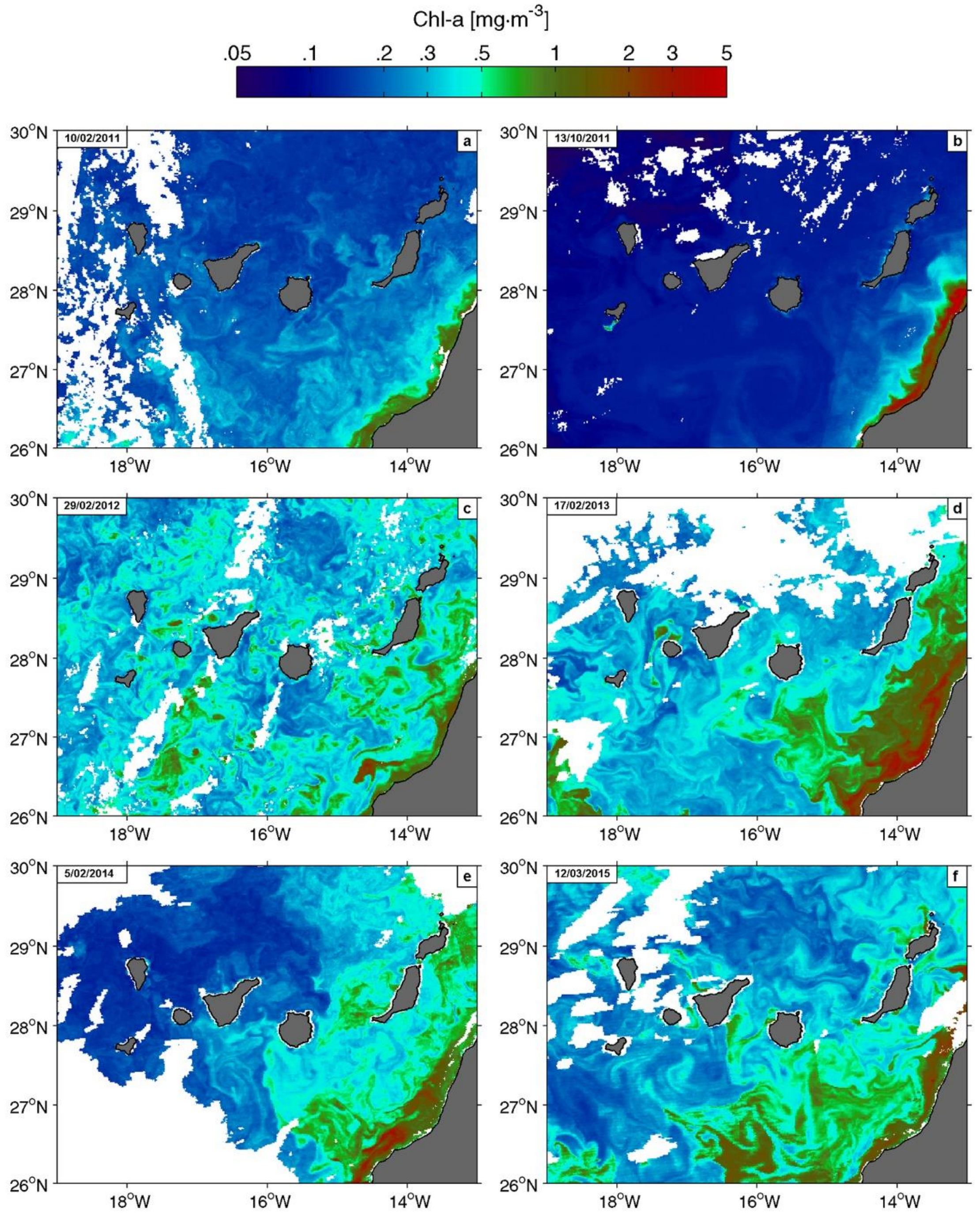

Supplementary Fig. S6. Remote sensing Chl-a (mg·m^{-3}) derived from ocean colour in the Canary Islands for selected days: a) 10/02/2011; b) 13/10/2011; c) 29/02/2012; d) 17/02/2013; e) 05/02/2014; f) 12/03/2015. Maps were generated using Matlab 7.14 R2012a (https://www.mathworks.com/products/matlab).
